# Complete Genome Sequence of the Hippuricase-Positive *Campylobacter avium* Type Strain LMG 24591

**DOI:** 10.1128/genomeA.01221-17

**Published:** 2017-10-26

**Authors:** William G. Miller, Mary H. Chapman, Emma Yee, Joana Revez, James L. Bono, Mirko Rossi

**Affiliations:** a Produce Safety and Microbiology Research Unit, Agricultural Research Service, U.S. Department of Agriculture, Albany, California, USA; b European Centre for Disease Prevention and Control, Stockholm, Sweden; c Meat Safety and Quality Research Unit, Agricultural Research Service, U.S. Department of Agriculture, Clay Center, Nebraska, USA; d Faculty of Veterinary Medicine, University of Helsinki, Helsinki, Finland

## Abstract

*Campylobacter avium* is a thermotolerant *Campylobacter* species that has been isolated from poultry. *C. avium* was also the second hippuricase-positive species to be identified within *Campylobacter*. Here, we present the genome sequence of the *C. avium* type strain LMG 24591 (=CCUG 56292^T^), isolated in 2006 from a broiler chicken in Italy.

## GENOME ANNOUNCEMENT

*Campylobacter* spp., primarily *C. jejuni* and *C. coli*, are commonly associated with acute bacterial gastroenteritis in humans (1), and transmission is often via contaminated poultry products (1, 2). In 2006, hippuricase-positive *Campylobacter* strains were isolated from poultry on three farms in Italy (3). Although initially identified as *C. jejuni*, based on their hippuricase activity, additional molecular and phenotypic tests identified these organisms as a novel species, termed *Campylobacter avium* (3). In this study, we present the first closed genome sequence of the *C. avium* type strain LMG 24591.

The genome of *C. avium* strain LMG 24591^T^ was completed using the Roche 454, Illumina HiSeq, and PacBio next-generation sequencing platforms, as previously described (4). Illumina HiSeq reads for strain LMG 24591^T^ were obtained from SeqWright (Houston, TX). The final coverage across the genome was 1,191×. The LMG 24591^T^ assembly was additionally verified using a bacterial optical restriction map (XbaI; OpGen, Gaithersburg, MD). Putative coding sequences (CDSs) were identified using GeneMark (5). Final annotation, including manual start codon curation, determination of homopolymeric GC tract variability, and the identification of rRNA- and tRNA-coding genes and pseudogenes, was performed as previously described (6).

*C. avium* strain LMG 24591^T^ has a circular genome of 1,738.6 kbp, with a GC content of 34.2%. The genome contains 1,645 putative protein-coding genes, 48 pseudogenes, 2 rRNA loci, and 3 putative genetic islands, with one encoding a partial type VI secretion system. Forty-four GC tracts of ≥8 bp were identified in the LMG 24591^T^ genome; 40 of these were determined to be hypervariable. No plasmids were identified in LMG 24591^T^.

Noteworthy in *C. avium* is the absence of the selenocysteinyl tRNA and genes encoding selenium-associated proteins, e.g., selenocysteine insertion proteins, selenoproteins, and selenoprotein-associated chaperones. The absence of selenium metabolism was reported previously for *Campylobacter lanienae* and related taxa (7). An ortholog of the *C. jejuni* fibronectin-binding protein CadF is also not encoded by *C. avium*. Because CadF is required for *C. jejuni* host cell invasion and colonization (8, 9), its absence might indicate reduced virulence in *C. avium*. Other proteins that are not encoded by *C. avium* include the ferredoxins FdxA and FdxB, methionine sulfoxide reductase (MsrA, MsrB, or MsrAB), and the globin Cgb, suggesting that, when compared to *C. jejuni*, *C. avium* might have a lower aerotolerance and is more sensitive to oxidative and nitrosative stress (10–12).

Prior to the identification of *C. avium*, *C. jejuni* was unique among *Campylobacter* spp. in its ability to hydrolyze hippuric acid (13). Hippuric acid (*N*-benzoylglycine) is cleaved by HipO (14) to produce glycine and benzoic acid, with glycine production measured by a simple colorimetric assay (15). Although we confirmed that *C. avium* is hippuricase positive, the *hipO* gene was not detected in strain LMG 24591^T^. Thus, it is likely that *C. avium* encodes an alternate hippuricase with low similarity to HipO. *C. jejuni hipO* contains a peptidase M20 domain and encodes a predicted zinc-dependent aminoacylase/carboxypeptidase. Analysis of the *C. avium* LMG 24591^T^ genome identified a candidate hippuricase gene with a similar domain structure, which we have termed *hipA*.

### Accession number(s).

The complete genome sequence of *C. avium* strain LMG 24591^T^ has been deposited in GenBank under the accession number CP022347.

## References

[1] Fitzgerald C 2015 Campylobacter. Clin Lab Med 35:289–298. doi:10.1016/j.cll.2015.03.001. 26004643

[2] Taylor EV, Herman KM, Ailes EC, Fitzgerald C, Yoder JS, Mahon BE, Tauxe RV 2013 Common source outbreaks of *Campylobacter* infection in the USA, 1997–2008. Epidemiol Infect 141:987–996. doi:10.1017/S0950268812001744. 22892294 PMC9151887

[3] Rossi M, Debruyne L, Zanoni RG, Manfreda G, Revez J, Vandamme P 2009 *Campylobacter avium* sp. nov., a hippurate-positive species isolated from poultry. Int J Syst Evol Microbiol 59:2364–2369. doi:10.1099/ijs.0.007419-0. 19620353

[4] Miller WG, Yee E, Chapman MH, Bono JL 2017 Comparative genomics of all three *Campylobacter sputorum* biovars and a novel cattle-associated *C. sputorum* clade. Genome Biol Evol 9:1513–1518. doi:10.1093/gbe/evx112. 28633450 PMC5499875

[5] Besemer J, Borodovsky M 2005 GeneMark: web software for gene finding in prokaryotes, eukaryotes and viruses. Nucleic Acids Res 33:W451–W454. doi:10.1093/nar/gki487. 15980510 PMC1160247

[6] Miller WG, Yee E, Chapman MH, Smith TP, Bono JL, Huynh S, Parker CT, Vandamme P, Luong K, Korlach J 2014 Comparative genomics of the *Campylobacter lari* group. Genome Biol Evol 6:3252–3266. doi:10.1093/gbe/evu249. 25381664 PMC4986449

[7] Miller WG, Yee E, Lopes BS, Chapman MH, Huynh S, Bono JL, Parker CT, Strachan NJC, Forbes KJ 2017 Comparative genomic analysis identifies a *Campylobacter* clade deficient in selenium metabolism. Genome Biol Evol 9:1843–1858. doi:10.1093/gbe/evx093. 28854596 PMC5570042

[8] Rubinchik S, Seddon A, Karlyshev AV 2012 Molecular mechanisms and biological role of *Campylobacter jejuni* attachment to host cells. Eur J Microbiol Immunol 2:32–40. doi:10.1556/EuJMI.2.2012.1.6. PMC3933988 24611119

[9] Ziprin RL, Young CR, Stanker LH, Hume ME, Konkel ME 1999 The absence of cecal colonization of chicks by a mutant of *Campylobacter jejuni* not expressing bacterial fibronectin-binding protein. Avian Dis 43:586–589. doi:10.2307/1592660. 10494431

[10] van Vliet AH, Baillon MA, Penn CW, Ketley JM 2001 The iron-induced ferredoxin FdxA of *Campylobacter jejuni* is involved in aerotolerance. FEMS Microbiol Lett 196:189–193. 11267778 10.1111/j.1574-6968.2001.tb10563.x

[11] Elvers KT, Wu G, Gilberthorpe NJ, Poole RK, Park SF 2004 Role of an inducible single-domain hemoglobin in mediating resistance to nitric oxide and nitrosative stress in *Campylobacter jejuni* and *Campylobacter coli*. J Bacteriol 186:5332–5341. doi:10.1128/JB.186.16.5332-5341.2004. 15292134 PMC490904

[12] Atack JM, Kelly DJ 2008 Contribution of the stereospecific methionine sulphoxide reductases MsrA and MsrB to oxidative and nitrosative stress resistance in the food-borne pathogen *Campylobacter jejuni*. Microbiology 154:2219–2230. doi:10.1099/mic.0.2008/019711-0. 18667555

[13] Fitzgerald C, Whichard J, Nachamkin I 2008 Diagnosis and antimicrobial susceptibility of *Campylobacter* species, p 227–243. *In* Nachamkin I, Szymanski CM, Blaser MJ (ed), *Campylobacter*, 3rd ed. ASM Press, Washington, DC.

[14] Hani EK, Chan VL 1995 Expression and characterization of *Campylobacter jejuni* benzoylglycine amidohydrolase (hippuricase) gene in *Escherichia coli*. J Bacteriol 177:2396–2402. doi:10.1128/jb.177.9.2396-2402.1995. 7730270 PMC176897

[15] Hwang MN, Ederer GM 1975 Rapid hippurate hydrolysis method for presumptive identification of group B streptococci. J Clin Microbiol 1:114–115. 1100648 10.1128/jcm.1.1.114-115.1975 PMC274963

